# Current progress in Structure-Based Rational Drug Design marks a new mindset in drug discovery

**DOI:** 10.5936/csbj.201302011

**Published:** 2013-04-02

**Authors:** Valère Lounnas, Tina Ritschel, Jan Kelder, Ross McGuire, Robert P. Bywater, Nicolas Foloppe

**Affiliations:** a CMBI, NCMLS Radboud University, Nijmegen Medical Centre, Geert Grooteplein 26-28, 6525 GA Nijmegen, The Netherlands; b Computational Drug Discovery, CMBI, NCMLS, Radboud University Medical Centre, Geert Grooteplein 26-28, 6525 GA Nijmegen, The Netherlands; c Beethovengaarde 97, 5344 CD Oss, The Netherlands; d BioAxis Research BV, Pivot Park, Molenstraat 110, 5342 CC Oss, The Netherlands; e Magdalen College, High Street, Oxford OX1 4AU, United Kingdom; f 51 Natal Road, Cambridge, United KingdomUK

**Keywords:** Rational drug design, virtual screening, protein kinase, G-protein coupled receptors, ligand binding thermodynamics

## Abstract

The past decade has witnessed a paradigm shift in preclinical drug discovery with structure-based drug design (SBDD) making a comeback while high-throughput screening (HTS) methods have continued to generate disappointing results. There is a deficit of information between identified hits and the many criteria that must be fulfilled in parallel to convert them into preclinical candidates that have a real chance to become a drug. This gap can be bridged by investigating the interactions between the ligands and their receptors. Accurate calculations of the free energy of binding are still elusive; however progresses were made with respect to how one may deal with the versatile role of water. A corpus of knowledge combining X-ray structures, bioinformatics and molecular modeling techniques now allows drug designers to routinely produce receptor homology models of increasing quality. These models serve as a basis to establish and validate efficient rationales used to tailor and/or screen virtual libraries with enhanced chances of obtaining hits. Many case reports of successful SBDD show how synergy can be gained from the combined use of several techniques. The role of SBDD with respect to two different classes of widely investigated pharmaceutical targets: (a) protein kinases (PK) and (b) G-protein coupled receptors (GPCR) is discussed. Throughout these examples prototypical situations covering the current possibilities and limitations of SBDD are presented.

## How drugs were discovered in the past

Historically, endogenous bioactive ligands were identified long before their cognate biomolecular receptor could be isolated and before their chemical structure was established [1–3]. Early information on ligand chemical structure was rudimentary and scarce compared with the many details provided by modern experimental chemistry and calculated wave functions. However, that early information often proved sufficient to infer knowledge and develop hypotheses on the physiological relevance of these ligands, even though the underlying physiological mechanisms were unknown.

The steroid hormones extracted from the bile acids at the beginning of the 20^th^ century is an emblematic example of this paradigm. In the 1920s and 1930s several Nobel prizes were awarded for discoveries connecting the steroids with the oxidation process in living cells, vitamins, sexual development and pregnancy, as well as relating the steroid hormones produced by the adrenal cortex with biological effects [4]. This led to the recognition of the polycyclic carbon framework of steroids. Using expert methods based on the stepwise pyrolytic breakdown of chemical structures, considerable efforts were spent to determine how the component rings of steroids were arranged. In 1932, the advent of X-ray diffraction was decisive in revealing directly the 3-dimensional arrangement of the atoms constituting the steroid scaffold, and the exact position and stereochemistry of the substituents [5]. Combining the structural details of steroidal ligands with biochemical knowledge showed how variations in the chemical groups substituted at specific positions around their scaffold were related to specific biological activity; hence allowing new medicines to be developed [4, 6].

Nowadays drug discovery is driven by biological targets, genetic studies, transgenic animal models, molecular biology, gene technology, and protein science. Hence, the structure of the target biomolecule is of great practical interest. With synchrotrons and fast computers, drug designers can visualize ligands bound to their target providing a wealth of details concerning the non-bonded interactions that control the binding process (Van der Waals repulsive and attractive forces, Hydrogen-bonds, salt-bridges, and mediation by water molecules and ions). Various computational techniques (visualization, docking, molecular simulations, quantum chemistry, *de novo* design) have been devised to score the ligand-protein interactions, including attempts to estimate the binding affinity of novel molecular entities with pharmacological activity.

Despite the availability of many co-crystallized ligand-receptors X-ray structures, as well as a flora of computational methods that can be accessed via sophisticated molecular modeling software, only part of the physical reality can be perceived and/or rendered by modern computer-based techniques, casting suspicion on the overall validity of the field [7, 8]. For example, the thermodynamics of the ligand-receptor association cannot be simply inferred from calculating close contact interactions, a situation which dramatically hinders scientific efforts toward truly effective rational drug design [7, 8].

In this minireview we examine the methodological trends that have emerged recently in the computer-aided molecular design of pharmacologically relevant ligands and how successful attempts were made to rationally combine X-ray, modeling and calculation techniques.

## The landscape of modern drug discovery

### The paradigm of probabilities in drug discovery

**Figure 1:**
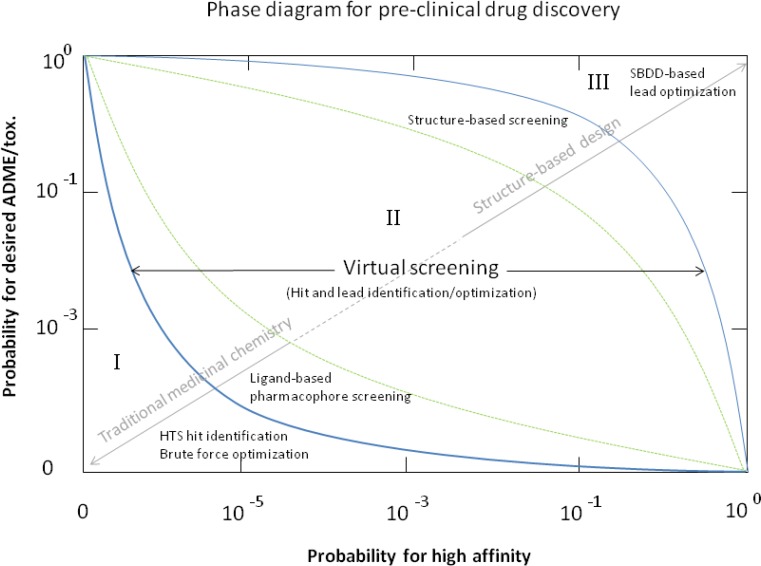
Idealized description of the probability landscape of the pre-clinical drug discovery process. From the identification of hit compounds with low to medium affinities to the optimization of lead compounds with high affinity and favorable ADME/tox, the process of preclinical drug development is a highly complex problem. Affinity and desired ADME/tox constitute two independent (orthogonal) dimensions that, unfortunately, cannot be easily optimized separately. Classical/brute force high-throughput identification and optimization of hits too often fails to provide compounds with both high affinity and adequate ADME/tox properties. This situation confines project outcome to a low-success probability pitfall represented by the bottom of the phase diagram (area I). Escape from this zone can be achieved with the help of a broad variety of virtual screening techniques encompassing pharmacophore-based techniques and structure-based techniques (area II). Ultimately, structure-based considerations can help drug designers guide a project towards a more productive area (III) of the phase diagram. Orders of magnitude for probabilities are only a rough guess and the frontiers between areas are schematic.

Are there more stars in the universe than possible organic molecules with a molecular weight < 600? Observation of the cosmos has led astrophysicists to map the universe and suggest that there are about 10^23^ stars gathered in 10^11^ galaxies [9]. In parallel, consideration of the real number of possible ligands has been the subject of savvy estimates [10, 11]. Complicating the matter is the fact that not all chemically plausible molecular structures might be synthetically accessible nor might they be affordable. Nevertheless, figures commensurate with the number of stars have been proposed. Neither the universe nor the ensemble of possible ligands can be explored systematically. The surrounding universe hints that extraterrestrial civilizations may exist, but the odds that a spaceship travelling for centuries at the speed of light may encounter one of them would remain so small that such enterprise would be doomed; a dreadful calculation which most science fiction aficionados are unaware of. A similar challenge is faced by high through put screening (HTS) widely used by the pharmaceutical industry in hit compound identification. A spokesperson from the industry modestly acknowledges this issue, skillfully admitting that: ‘the discovery task has shifted somewhat during the past few years from simply identifying promising leads to the added proviso that dead-end leads should be eliminated from consideration as early in the process as possible’ [12, 13]. In other words, the leads supplied by classical chemistry optimization rounds performed around HTS-supplied hits are often difficult to transform into drugs. Optimization of binding affinity in isolation by traditional medicinal chemistry methods leads to poor ADME/tox properties through effects such as the inclusion of bulk to ligands, excessive functionalization, growth of hydrophobic groups and/or selection of functional groups with supposedly known ADME/tox liabilities [14]. They are too few leads, they are not diverse and more importantly, HTS provides no information at all about the way they interact with the target receptor, thereby precluding efficient optimization (Figure 1). The screened collection tends to represent what happens to be available in a particular organization, instead of rationally selected chemotypes. HTS favours quantity over quality and insight, and results in large amounts of data of dubious quality, which requires much time and effort to be analyzed. One could argue that the vast resources invested in HTS could have been used more productively to characterize how leads interact with the target receptor, to inform their efficient optimization.

In response, many research groups throughout the world have dedicated efforts toward structure based drug design (SBDD) and virtual ligand screening (VLS), as the most scientifically promising approaches to identify ligands for pharmaceutical targets [15–20]. SBDD provides insight in the interaction of a specific protein-ligand pair, allowing medicinal chemists to devise highly accurate chemical modifications around the ligand scaffold, whereas the strength and versatility of VLS are self-evident as it can be used to filter compounds to be assayed based on any rationale that can be expressed in terms of molecular properties (pharmacophores and/or physico-chemical descriptors). Here, we should point out that docking programs can be used to filter libraries of compounds [21]. However, it remains extremely complicated to rationally analyze docking results for libraries containing chemically diverse compounds [21]. The lack of reliable of scoring functions across heterogeneous chemical classes and the frequent impossibility to predict the correct binding modes has generally cast doubt over docking as a genuine screening method. We shall see in further sections how one may combine VLS with SBDD and how docking can be efficiently used as a virtual screening tool as well in specific circumstances. SBDD offers distinctive advantages compared with the classical ligand-based techniques such as the comparative molecular field analysis (CoMFA) and pharmacophore-based techniques, based on only ligand information, that have well known limitations [22]. Another crucial advantage is that electronic libraries can cover already existing compounds as well as compounds that have not been synthesized yet but are chemically relevant.

SBDD can be broadly envisioned as one intrinsic component of rational approaches based on screening assays that are more adapted to drug discovery than HTS. For instance, fragment-based drug design (FBDD) has emerged as a very powerful means to reverse the HTS probability paradigm by exploiting molecular fragments with molecular weight < 150-250 [23, 24]. These fragments can be selected based on adequate physico-chemical properties, collected in libraries, assayed and, using SBDD-derived information, reassembled into larger molecules with improved drug like characteristics [25–27]. FBDD is a genuine branch of rational drug design for which all the principles we discuss in this minireview are valid and can be used. We refer the reader to recent reviews on FBDD [28, 29, 30].

SBDD can reveal important information on the structure activity relationships of a chemical series, especially in the lead optimization phase, when very accurate modifications are needed to adjust an ADME/tox profile while maintaining binding affinity. Target selectivity is another issue where SBDD may be vital, especially when dealing with large classes of pharmaceutical targets SBDD requires, however, that an X-ray structure be available for the pharmaceutical target, or that a ‘reasonably’ valid homology model has been constructed. We shall illustrate in the next sections what reasonable means. Knowledge of the target structure plays the same role as boundary conditions in physical sciences, providing indications for instance on the maximum possible volume that the ligand can occupy, and the particular loci and orientations where hydrophobic and/or hydrophilic interactions can be engaged.

### Is there a missing link in SBDD?

Ligand-based approaches (e.g., CoMFA and pharmacophore-based methods) are intended to capture and/or exploit a remarkable pattern detected predominantly from the empirical superposition of a set of experimentally identified binder and non-binder compounds [31, 32]. In this respect, only the first-order/gross characteristics of the ligand-receptor recognition and binding pattern are taken in consideration. With these techniques virtual library screening tends to retrieve large sets of compounds (hundreds to thousands) that are usually further filtered based on empirical considerations, excluding compounds that are not drug-like and relying on ‘luck’ (see previous section) to identify new binders within the remaining compounds.

In contrast SBDD aims to provide a richer picture of the ligand receptor interactions. However, SBDD has developed more slowly. Many X-ray structures of pharmaceutical targets co-crystalized with their natural ligands and/or inhibitors are publically available, however many more have been resolved by the pharmaceutical industry without being published [22]. Thus, it is difficult to estimate the extent to which these data have contributed in conceiving new drugs. This situation has hindered a fair evaluation of the true potential of SBDD by the scientific community and the merit of SBDD may not seem as widely praised as the ligand-based techniques. In many instances, at conferences and during informal discussions among experts, the benefit of SBDD in drug design remains a controversial issue [7]. For many drug designers, the lack of reliability in predicting affinities constitutes what could be referred to as the ‘missing link’ which undermines effective SBDD. But, if we do really believe that is the problem then we should simply resign as rational drug designers as there is no obvious way the accuracy gap will be bridged [33].

Intuitively, the difficulty of establishing the limit of applicability of the methods maybe more troubling to the productive development of SBDD than inaccuracy in energetic calculations. The 25^th^ anniversary issue of JCAMD has published a series of articles that all describe one or several aspects related to this concept. Quoting one of them seems appropriate: ‘Like poor mule drivers, we have a tendency to push our methods beyond their limits’ [7]. To address this misbehavior we have to realize that the time scale required by rational SBDD contrasts with the state of permanent urgency that characterizes the pharmaceutical industry in its quest for introducing new drugs to the market [34]. In this setting, a lead optimization program hardly lasts 12 to 18 months, a time too short to gather all experimental information and model validation necessary for detailed SBDD. Pharmacophore-based techniques are well adapted to this limit since they require far smaller time overhead than SBDD to establish a validated albeit crude model of receptor-ligand interaction [35]. But the rewards of SBDD are potentially greater compared with those techniques that have proven their paucity in providing sufficiently accurate rationales, especially in the lead optimization phase where not one but several properties must be optimized together.

### We need to keep an eye on the components of the free-energies of binding!

Despite considerable progress achieved in structure-based virtual ligand screening and computer-aided drug design [36–43], the reliable and practical prediction of binding affinities remains an elusive holy grail [44–51]. Even when knowing the binding mode of a ligand to its receptor, its binding affinity cannot be inferred reliably although, paradoxically, it can be measured highly accurately with titration calorimetry and surface plasmon resonance [52–54].

The affinity of the ligand for its receptor is dictated not only by the enthalpic contribution arising from close range physical interactions (Van der Waals interaction, hydrogen bonds, ionic pairing) but also by an entropic contribution that is a measure of the overall system disorder. In biology though, the concept of disordered systems is difficult to comprehend and is sometimes counter intuitive, as a ligand bound to its receptor may seem inherently a more ordered state than the unbound ligand and receptor when this is not necessarily the case [8]. Indeed, the entropy penalty resulting solely from the reduction of the overall body translational and rotational freedom of the ligand and receptor may amount up to 15 kcal/mol while the entropy penalty due to the reduction of rotational freedom is estimated to be ca. 0.6 kcal/mol per rotatable bond [53, 55]. Upon binding, favorable interactions with the receptor compete with these unfavorable energy terms. In addition, the disruption of ordered clusters of water molecules around the ligand hydrophobic moieties, a process which causes a gain of disorder in the solvent, contributes favorably to the stability of the ligand-receptor association [56, 57]. Experimental measurement showed that increasing the nonpolar surface area of an aliphatic alcohol by inserting a CH_2_ group has an unfavorable entropic contribution of +1.7 kcal.mol^−1^ to the free energy of transfer in aqueous solution, which is only partly compensated by an enthalpic contribution of -1.3 kcal.mol^−1^ [58]. The result is in an overall unfavorable free energy of 0.4 kcal.mol^-1^. If not counterbalanced by other effects, a change of this magnitude in the hydration energetics of a ligand corresponds to an increase by a factor of 2 in its measured binding affinity (IC50); and we know that optimization of a lead often operates in a range less than 1.4 kcal mol^−1^ (i.e., less than a factor of 10 in IC50s) [7]. Increasing the hydrophobicity of a ligand is a strategy often used in drug discovery. However, this change has to be accommodated by the receptor and the ligand must remain a potential drug; ligands with excessively increased hydrophobicity have poor absorption, distribution and off-target toxic binding properties [14, 59]. Efforts to gain insight on how different chemical groups branched around a same scaffold may influence binding affinity are hampered because the experimental evaluation of the enthalpy (∆H) and entropy (T∆S) of binding (with ITC) are large quantities that compensate each other while having error bars exceeding 1.4 kcal mol^−1^, which is uncomfortably large in comparison to the overall binding energies [60]. In standard assays, however, the error in the binding free energy is usually in the range +/− [0.1 - 0.5] kcal/mol only, allowing results to be more readily exploited in drug design, though deeper insight in the binding process is limited.

An often neglected aspect in rational drug design is the ubiquitous presence of water inside and around the receptor binding cavity and in some instances, the free-energy cost of displacing ordered or partially ordered water molecules involved in the residual solvation of active site residues should be considered carefully [61–64]. Computational methods are being devised to calculate the energy cost of displacing clusters of rotationally and translationally hindered water molecules present in binding site cavities accessible to water [65–70].

When a ligand binds to a protein, water molecules surrounding the ligand and protein need to rearrange. These rearrangements contribute to the binding affinity of the ligand. Most water molecules building the hydration shell around the ligand and in the protein active site need to be either removed or displaced. Paying attention to the thermodynamics of these phenomena, for which no relevant experimental data are available, has been done in early theoretical studies of protein folding and protein association [71]. Very recently, molecular dynamics simulation (MDS) has further contributed to our understanding by showing that water molecules in the first hydration layer around a receptor surface are less thermodynamically stable than bulk water [61]. The process of transferring water from the bound state to bulk solvent is associated with a favorable free energy [61, 64, 65]. This, however, does not hold for water molecules that are deeply buried and tightly bound in crevasses at the surface or inside the active site of proteins. The maximum entropy change of releasing such a water molecule into bulk water is evaluated around 2 kcal.mol^−1^; a gain which is unfavorably outweighed by the loss of enthalpy due to the breaking of hydrogen bonds with the receptor [63]. Because they are highly ordered (immobilized), these water molecules are seen in X-ray crystal structures, and in many cases ligand binding is often mediated by such water molecules [66]. Attempting to displace them with experimental ligands may not only be difficult to achieve but also have unpredictable unfavorable effects [63, 72, 73].

However, when a ligand is binding to a hydrophobic cleft at the surface or inside the protein the enthalpic contribution to the free energy of binding will increase because of favorable interactions between the ligand and protein. Simultaneously, water molecules that occupy the hydrophobic cleft will be released into the solvent, resulting in a favorable increase in entropy [61].

**Figure 2:**
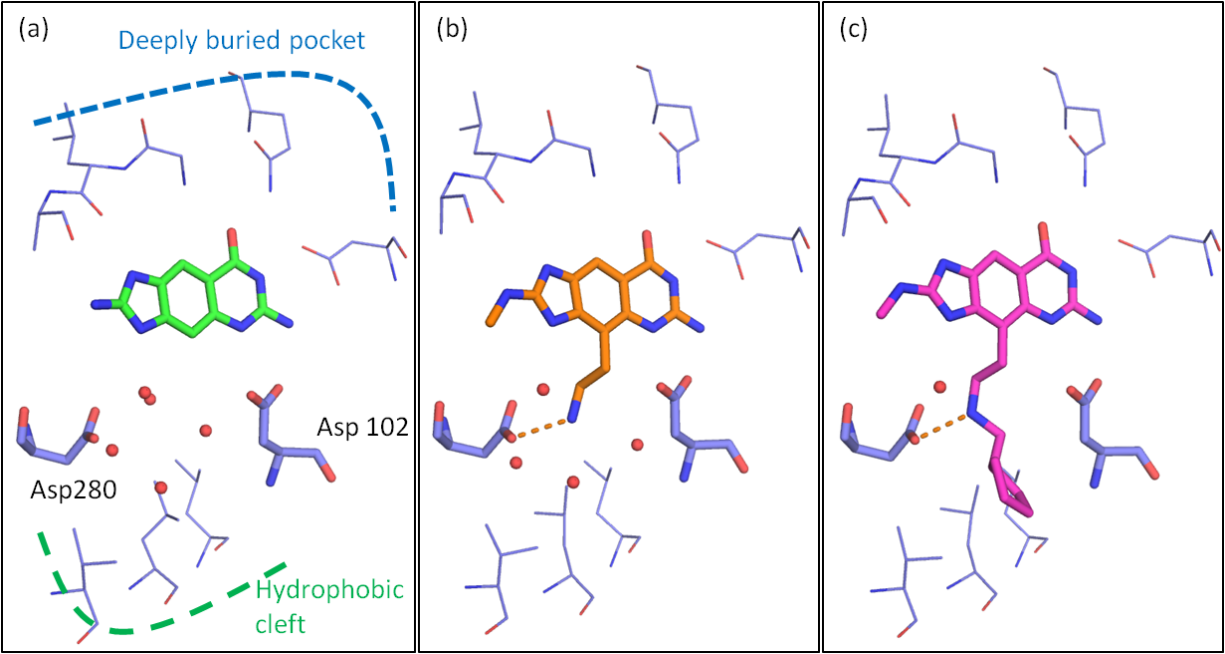
Example of how SBDD can be applied to displace highly ordered (immobilized) waters in the active site of an enzyme: (**a**) X-ray structure of *lin*-benzogunaine in complex with TGT with a binding affinity 58 nM; (**b**) an amine substituent that could mimic the water molecules while maintaining the same level of affinity for TGT (55 nM) was introduced; (**c**) substitution of the amine to occupy the small hydrophic pocket with a cyclohexyl moiety increased the binding affinity significantly (2nM).

The discovery and optimization of inhibitors for the enzyme tRNA-guanine-transglycosylase (TGT), an enzyme from pathogenic Shigella bacteria, is an example where desolvation of the ligand and protein has a significant influence on the binding affinity of the inhibitors [62, 74]. The tRNA binding site of TGT consists of one deeply buried pocket surrounded by smaller, solvent exposed pockets (Figure 2) in between two negatively charged catalytic aspartates. Using classical SBDD approaches, attempts were made to substitute the *lin*-benzoguanine scaffold with a scaffold that occupies the hydrophobic cleft situated below the two aspartates to improve potency. However, occupation of this hydrophobic cleft did not result in the expected gain in binding affinity.

In the crystal structure of *lin*-benzoguanine in complex with TGT, an ordered solvation shell solvating the two proximal charged aspartates was discovered [75]. Functional groups such as amine substituents that could mimic the water molecules were introduced. Comparing the binding affinity of the scaffold *lin*-benzoguanine (Figure 2a, 58 nM) with the unsubstituted amine (Figure 2b, 55 nM), binding affinity could be maintained (cost neutral) while restoring the favorable interaction provided by the displaced network of water hydrating the aspartates. Hydrophobic substitutions on the amine (with for example a cyclohexyl moiety) increased the binding affinity significantly by accessing a lipophilic pocket below the catalytic aspartates (Figure 2c, 2nM).

### Homology models to generate and verify binding mode hypothesis

The choice of the template(s) used for the homology model construction may lead to Cα positional variations of the order of 1 to 3 Å (or more) in the active sites of enzymes, depending on the percentage of sequence identity shared with the elected template(s) [76, 77]. Such variations have to be appraised during a model construction because they may lead to substantial side chain displacements in the final model. Various techniques can be used to mitigate this problem: the use of multiple templates or a mixed template etc. In principle, homology modeling software can be used to optimize side chain conformations and the non-bonded interactions between them by simulated annealing, conditional probability distribution, or potential of mean force [78]. At this stage there are no safe recipes to apply but careful physical insight along with verification that hydrogen bond networks and hydrophobic residue packing statistically conform with the knowledge-based characteristics extracted from the X-ray structures [79]. Conserved interactions between particular residues (hydrogen bonds, ionic bridges,….) in the active site may be also used as a guide to adjust the model during its elaboration [80, 81]. One has to avoid the pitfall of model overoptimization that might actually drive the model away from physical reality. At the same time the model should be consistent with respect to most (if not all) available experimental data [77, 79]. Failing to abide by this necessity inevitably leads to a high risk of project failure in a later phase when the simultaneous optimization of other properties, such as absorption, metabolism, excretion and toxicity (ADME/tox), must be performed for a lead compound to become a real drug, while retaining a high affinity and selectivity for the pharmacological target. The next sections illustrate with recent examples that this approach can be effectively put in practice; successfully combining two or more modeling techniques together (Homology Modeling, Docking, MDS,…).

## Different types of ligand-receptor complexes and different challenges

There are a plethora of X-ray structures of receptor-ligand complexes for several important families of drug targets, including proteases, HIV reverse transcriptase, nuclear receptors (NR), protein kinases (PK), and even RNA targets [82]. Structures have also started to become available for some G-protein coupled receptors (GPCR). Some of these receptors are already privileged targets for SBDD (e.g., NR, PK) and some others might also provide other avenues for SBDD in due course (e.g., GPCR, RNA) [83, 84]. This wealth of structural information will continue to increase, also as a result of the structural genomics initiatives [85].

**Figure 3:**
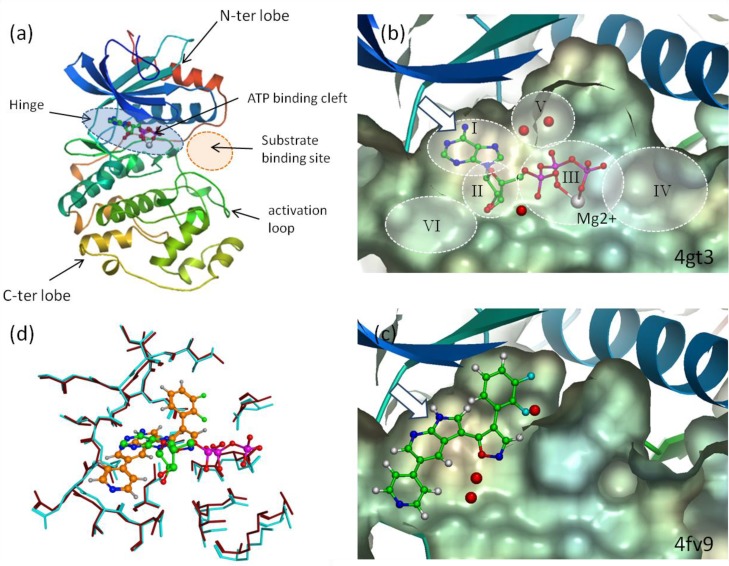
(**a**) Ribbon representation of Erk2 kinase in its activated form with ATP bound (pdb entry 4gt3). The ATP binding cleft has a large solvent exposure and can be decomposed into several compartments or pockets (I to VI)(b). The average rate of conservation for the residues lining it is only 51% [80]. Together these observations allow the design of a large variety of selective inhibitors. Subareas (**b**) and (**d**) show clipped views of the ATP binding site seen from the top, for two X-ray structures of Erk2 with, respectively, ATP (pdb entry 4gt3) and the synthetic ligand E71 (pdb entry 4fv9). Note that highly ordered water molecules (red spheres) mediate the binding in both cases. The synthetic ligand is anchored to the hinge residues via the same two hydrogen bonds (bidentate motif; white arrow) as does ATP. However, it is oriented differently and expands perpendicularly to the cleft main axis, occupying the buried pocket (V) on one side and extending to the outer part of the cleft (VI) on the other side (**c**). Despite considerable difference in the chemical structures and binding modes of ATP and E71 the deviation between the backbone and side chains atoms of the two X-ray structures is small (<1Å) (4fv9 brown and 4gt3 cyanide blue) (**d**). Although it is not true in 100% of cases, conformational invariance of the active site residues can be reasonably assumed in many SBDD-based drug design projects.

**Figure 4:**
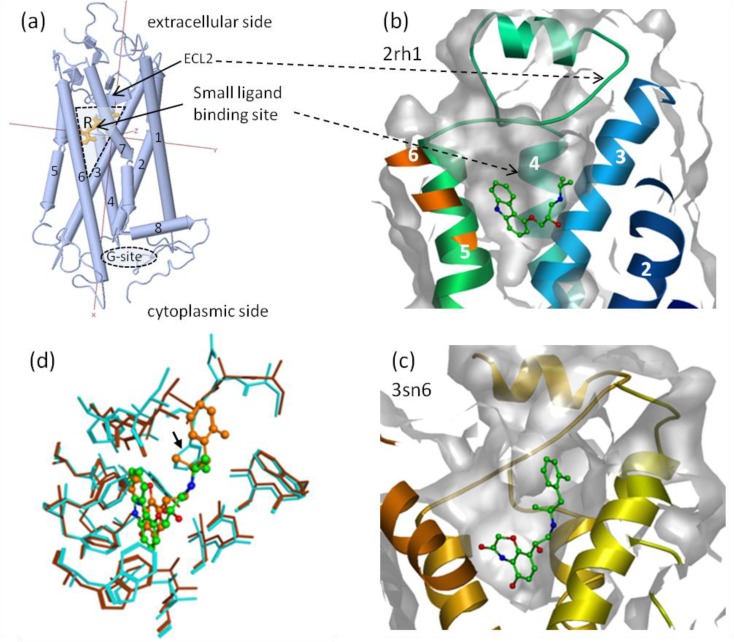
(**a**) Cartoon representation of X-ray structures of bovine rhodopsin, representative of class A GPCRs: with retinal (R) in the cis form bound before it has absorbed light (inactivated form, PDB* entry 1u19; side view (**a**)). GPCRs are made of a bundle of 7 alpha helices (numbered from 1 to 7 in view (**a**) and (**b**)) topped by a large -pleated sheet structure resulting from the packing of two helix connecting loops. One additional helix (8) located at the interface between the cell membrane and cytosol has a regulatory role in deactivating the receptor. A major evolutionary constraint on GPCR function is to bind G-proteins in a large crevasse at their cytoplasmic side (G-site) [128]. The structural change that leads to GPCRs activation (G-protein coupling) is a general mechanism controlled by an extremely broad array of endogenous effectors (modulators) ranging from large structured peptides such as chemokines and peptide hormones to low molecular weight ligands that bind in the cone-shape cavity buried inside the 7-helix transmembrane bundle near the extracellular side of the receptor. Illustration of GPCR plasticity is given with: (**b**) zoomed side view of X-ray structure of 2AR in complex with the antagonist (S)-Carazolol (2AR inactive state, PDB entry 2rh1); (**c**) X-ray structure of 2AR in complex with a high affinity agonist (BI-167107) (2AR-Gs protein active complex, PDB entry 3sn6); (**d**) overlay of the side views (**b**) and (**c**) showing residues in close contact (< 5 Å) with the antagonist (cyanide blue) and the agonist (brown). Positional shifts of side-chain and main-chain atoms highlight the plasticity of the small ligand-binding site; the side chain of Phe 193 (black arrow) was too disordered to be resolved in the agonist bound structure. (*see reference [82])

When exploiting structural information, drug designers are confronted with situations that bear similarities within the same class of targets. This gives rise to what we refer to as prototypical situations of ligand-receptor recognition, but not simply ‘routine situations’ because each project requires its own logical development. Different types of receptors possess unique challenges for SBDD. We present two example systems here:

- Protein kinases (PK) (Figure 3): multidomain proteins with complex catalytic domain ligand binding sites (large cleft between two domains); ATP is the natural ligand; weakly bound water and Mg or Mn ions mediate ATP binding; the vast majority of PK inhibitors target the ATP binding site; selectivity is a crucial issue that requires SBDD-based considerations; a well defined purine binding motif acts as an anchor; possibility of allosteric binding/inhibition; activation occurs via loop phosphorylation; many crystal structures of complexes exist and homology models can be routinely constructed; PKs are well suited for SBDD.
- GPCR (Figure 4): 7-helix bundle transmembrane assembly; very versatile binding modes, deeply buried inside the transmembrane region; possibility of allosteric binding, agonism and antagonism effects, selectivity is an issue; binding cavity is very complex and topped by large highly flexible loops; mechanism of activation/inhibition requires more in depth understanding; not yet routinely amenable to SBDD; more X-ray structures and binding experiments combined with mutational data are expected to clarify this mechanism.

### Designing selective ATP competitors to modulate protein kinase activity

Although PKs constitute only 2% of the human genomes they represent ∼ 25% of putative drug targets [86, 87]. They are multidomain proteins that intervene in many biological processes, especially signal transduction. Their catalytic domain (called kinase domain) chemically modifies other proteins by transferring the γ-phosphate group from a nucleoside triphosphate (usually ATP) to the hydroxyl group of specific residues such as serine, tyrosine, threonine, and sometimes to histidine. Deregulation of these processes is implicated in many physiological disorders, including inflammatory diseases and cancer [88, 89]. Hence, protein kinases are prominent drug targets as reflected by the number of deposited patents [90].

Since the first X-ray structures of the kinase domain appeared, it was understood that the kinase domain had a highly conserved fold across all members of the superfamily [91–93]. This fold comprises two connected domains that form a cleft where ATP binds, adjacent to a shallow substrate binding region (Figure 3). The fact that proteins can be categorized in a large superfamily sharing a common fold has far-reaching practical impact in drug design because it is expected that a substantial amount of information gleaned from one kinase can be transferred to other ones. Indeed, the conserved natural ligand (ATP) of PKs means that their binding site is also somewhat structurally conserved, which was initially perceived as a drawback preventing the discovery of selective ATP-competitive inhibitors. Competing with ATP was also deemed difficult since it is abundant in cells [94].

On the other hand, this situation has the advantage that structural models may be obtained by homology (comparative) modeling for most kinase domains [80]. This should signify a successful future for SBDD, as long as, modelers assess the potential weakness of their model prior to its use and bear in mind a number of practical limitations that continue to hamper the development of protein models constructed by homology modeling. First, the opening of the two lobes that sandwich the ATP binding site can vary by a few degrees, due to the flexibility of the hinge region connecting them, causing substantial displacements (up to several Å) in the relative positioning of the residues lining ATP at the other extremity of the binding pocket making the ideal alignment of structures often ambiguous [92, 95]. Second, as happens almost systematically in homology model construction, the multiple sequence alignments used for selecting templates cannot be 100% ascertained because of gaps and insertions, especially with superfamily members characterized by a low sequence identity (< 40%), even around the active site, and even when multiple templates are used and class-specific information (position-specific sequence conservation) is implemented in the sequence alignment [76, 96, 97]. Third, despite elaborate algorithms using conformational propensities derived from X-ray structures, it is difficult to accurately assert which residue side chain conformations are physically relevant for each particular ligand-receptor association and the positions of the residue side chain lining the active site of a homology constructed model remains questionable [79, 98–100]. Fourth, the possible effect due to the presence of mediating water molecules and/or counter ions inside the active site is difficult to predict, except for the few conserved (tightly bound/structural) examples that are inferred from X-ray structures [101]. Many research groups have developed *state-of-the-art* semi-automated methods that allow experienced drug designers to construct knowledge-based homology models where issues related to limitations 1, 2 and 3 can be tackled in a ‘fairly’ rational way in many cases and, less frequently, in an evidence-based way [79]. However, the way these models are built cannot be generalized and their validity must be assessed on a per individual project basis due to the conditions and specific interactions involved in ligand binding being extremely variable.

A kinase structural database (Kinase Knowledge Base, KKB) has been recently developed that covers about 148 different kinase domain structures that have been deposited in the Protein Data Bank (PDB) and which represents about 20% of all kinase domains in the human genome [80]. Using a carefully selected set of 20 non-redundant X-ray structures overlaid with a protein structure alignment tool a structure-based sequence alignment of the 594 members of the human kinome has been generated [102].

This work opens the possibility for drug designers to benchmark their homology model construction methods. For instance, they can select a dozen kinases out of the 148 X-ray structures and reconstruct them by homology based on the kinome-wide sequence alignment provided in the KKB database. The comparison between the reconstructed set and the X-ray structures can provide drug designers valuable feedback to test their methodology.

The analysis of sequence conservation for active site residues shows the residue variability and character (hydrophobic, flexible, polar, aromatic, positively or negatively charged, and mixed) at every particular position in the ATP binding pocket [80]. This high variability together with the finding of kinase inhibitors that have a high affinity for a relatively reduced subset of kinases enables the SBDD approach (Figure 3) [102]. We shall show how in the detailed analysis presented in the next sections.

## The combined techniques approach

### Docking-based spatial superposition of ligands to produce synergy with QSAR

Accurately conducted docking studies can be used very efficiently to complement QSAR techniques such as CoMFA and comparative molecular similarity index analysis (CoMSIA) which crucially rely on how compounds are overlaid [103]. However, the validity of the docking methodology must be established by successfully reproducing the binding modes of some of the ligands belonging to the set of studied derivatives and for which X-ray structures are available in the PDB. The remaining compounds are docked following the same docking protocol. The purpose of docking is to provide a rationally derived structural alignment (spatial superposition) of the studied set of compounds, that in turn can be used to derive a predictive QSAR model based on molecular descriptors including steric, electrostatic, hydrophobic, and hydrogen bond donor fields as well as PSA and MLogP.

We see how a multi-stage approach and the use of two computational methods can complement each other and compensate for the respective weaknesses of each method, thus creating a synergy in SBDD. Docking scores are unreliable, and most QSAR techniques cannot be applied without either knowledge or a strong hypothesis of the bound conformations of the ligands and spatial alignment [104–106]. But if the preferred binding mode of chemically related compounds can be inferred, docking can provide the correct ligand conformation and an accurate structural alignment that is required to build a predictive QSAR model [107–109].

### Scaffold-based virtual ligand screening

Acquiring accurate knowledge of the binding modes for identified chemical classes of compounds is such a stringent issue in SBDD that strategies have developed to reverse engineer the problem and try to identify proto-ligand scaffolds with adequate affinity and physicochemical properties selected from the molecular characteristics of known ligands. This approach requires the assay of specific subsets of compounds extracted from specialized libraries containing relatively large molecular fragments within a molecular weight range of 150 – 250 Da. For instance in the case of protein kinases, prototypical scaffolds targeting the ATP binding site are preferably flat and contain at least two conjugated ring systems which can engage bidentate hydrogen bonding interactions [18, 110, 111].

Once proto-ligand scaffolds that compete with ATP have been identified in biochemical assays, they can be co-crystallized with the target enzyme and their binding mode determined by X-ray crystallography. They are particularly well suited for SBDD. Virtual libraries can be subsequently constructed by identifying the substitution positions best suited to expand the ligand around the scaffolds. Docking can be used to select the putative ligands that best fit the identified binding modes and that provide additional complementarity with the residues lining the binding site [111].

This approach is particularly well suited for PKs because the ATP binding cleft is large and open which allows for multiple possibilities to expand the ligand scaffold in various directions to search for selective interactions (Figure 3) [111]. The concept of scaffold-based series can be implemented in SBDD projects with a great deal of variation and prior screening in biological assays might not always appear necessary. For instance, a docking study performed on a series of PKs has shown that docking large sets of ligands that share the same scaffold is statistically more likely to retrieve the correct class-specific binding mode within the top clusters of docked poses than clustering 100 poses generated from a single ligand [112].

## SBDD with GPCRs

GPCRs (Figure 4) are important pharmaceutical targets for the treatment of many physiological disorders including some inflammatory diseases, neurotransmission and sensory perception impairment, diabetes, cardiovascular defects, AIDS and some cancers. At least 40% of the currently marketed drugs target GPCRs [113]. In the past decade, groundbreaking X-ray structures have been solved for an increasing number of class A GPCRs. The first one was the bovine rhodopsin receptor X-ray structure that has served from that time as a useful template for homology modeling of other GPCRs to replace the old bacteriorhodopsin based models. Nowadays X-ray structures are available for the adrenergic β1A and β2A, histamine H1, adenosine A2A, dopamine D3, muscarinic M2 and M3, opioid kappa and mu, CXCR4 receptors, etc., most bound to low molecular weight antagonists. These structures have helped assess the many ‘common mistakes’ that have crippled early GPCR models [114, 115]. It is fantastic that the importance of all this work starting from molecular cloning to X-ray structure was recognized by a Nobel Prize awarded in 2012 for the X-ray structure of the β2-AR-Gs protein complex [116, 117]. But the early models had served already as valuable 3D alignments that stimulated our thoughts on how ligands might bind, and have led to many successful suggestions for site-directed mutagenesis experiments to study ligand binding. Implementing valid SBDD considerations within drug design projects targeting GPCRs still remains difficult due to limited insights as to how ligands interact with them and modulate their activity [118].

Many experimental observations for rhodopsin have shown that residues from the second extracellular loop (ECL2) and located in the cone-shape cavity inside GPCRs interact with ligands to trigger the outward tilt of transmembrane helix 6 (TM6) relative to TM3 and TM5 [119]. This movement induces a concerted rearrangement of the whole helix bundle that conveys the activation signal to the intracellular side of the receptor, allowing G-protein coupling to the activated form of the receptor (Figure 4) [120–122]. This mechanism is thought to be general across class A GPCRs [123]. Both the activated and inactivated states are assumed to exist in vivo as an equilibrium governed by specific sets of amino acids acting as ‘micro-switches’ and conserved within class A subfamilies [124–128]. By interacting with these residues, agonists and antagonists stabilize one state or the other. Although the mechanism is general, it is modulated by different amino acids across subfamilies and subfamily members [129]. In 2007, the X-ray structure of the β2-adrenergic receptor proved that the spatial arrangement of the transmembrane helices relative to each other is highly similar to that of bovine rhodopsin; which was encouraging for homology modelling and SBDD [130]. However, the conformation of ECL2 was entirely different between the two structures.

Compared with other classes of receptors such as PKs and NRs the number of elucidated X-ray structures of GPCRs bound to small compounds is still too small to safely extrapolate to other classes of ligands in SBDD [131]. However, the number of GPCR X-ray structures is growing rapidly. Already, they have helped validate the use of docking and virtual ligand-screening (VLS) applied to GPCR crystal structures [131–133]. Until recently, the GPCR conformational plasticity was thought an obstacle for SBDD of agonists [134, 135]. Several X-ray structures of agonist-bound complexes have been produced in 2011 for β1AR, β2AR and A_2A_AR and more structures are expected in the near future. These structures will provide an improved 3D framework for the rational design of agonists and antagonists, illustrating the utility of modeling broader structural changes than only those limited to the residues lining the binding site pocket [120, 134].

The elucidation of the X-ray structure of bovine rhodopsin in 2000 put GPCR homology modelling on a reasonable footing, assuming a high overall structural conservation across members of the superfamily despite their very low sequence identities [136]. GPCR homology models of improved quality can now be prepared routinely thanks to database such as GPCRDB and GPCR-SSFE constructed in conjunction with the structural elucidation of several other class A GPCRs [114, 134, 135–140].

So far, antagonist/agonist ligands have been mostly identified using ligand based approaches consisting of filtering libraries of commercially available compounds with pharmacophore models [141–143]. Nevertheless, carefully constructed homology models of rhodopsin-like GPCRs are increasingly reported, with the concomitant use of docking, which allowed enrichment of screened compounds with actives with rates similar to those obtained with the ligand-based pharmacophore approaches [144–154]. Crucially, the advantage of structure-based screening would not be only in an improved success rate but also in providing: ‘useful information on the structurally vital residues involved in binding, and their unexplored potential for the future development of potent receptor antagonists’ [145].

Hence, the design of GPCR modulators will ultimately require the ability to make a ligand have a defined functional profile in terms of agonism/antagonism. This assumes that the micro-switch residues with which ligands engage are correctly identified and that these interactions are accurately modeled [127–129]. A recent multiple sequence-alignment based bioinformatics analysis including 1664 receptor-ligand pairs comprising 767 full agonists, 184 partial agonists and 713 antagonists illustrates how large scale data analysis can provide a deeper understanding of the GPCR activation mechanism [118]. This set covered 100 class A GPCRs belonging to 30 subfamilies. Residues at relative positions determined by a multiple sequence alignment were correlated with ligands’ physico-chemical descriptors and activities. Such approaches provide information that, in principle, can be exploited in combination with homology models to generate many working hypotheses [155, 156]. How can SBDD help in sorting and filtering them to select the relevant ones? In the coming years, drug designers, chemists and biologists will be exposed to a daunting combinatorial explosion of hypotheses, with the cumbersome task to explore them by trial and error [144]. We assume that more integrated multi-software SBDD systems combining state of the art modeling, bioinformatics procedures and bioassay results including site-directed mutagenesis will assist drug designers in these arduous tasks [144, 155, 157, 158].

## Concluding remarks

Over the 35 years that have followed the first published work describing SBDD in 1976, computer aided molecular design and SBDD has surmounted several hurdles and has played a key role in the development of several marketed drugs [22, 159]. The proof of concept is now well established for SBDD, but cultural shifts are still needed to accept and implement this strategy broadly [159].

Modern genomic investigations and translational research have identified a wealth of new targets. Certainly, in the past decade the number of targets has increased more rapidly than the discovery of new drugs. The plethora of new pharmacological targets must be accompanied by the discovery of new drugs and active classes of ligands in a sustainable manner; otherwise the continuous health care improvement that has characterized the 20^th^ century will fade. The industry is highly profitable thanks largely to revenue from public health schemes and insurance contributions, yet the high costs and risks associated with new drug discovery have been used to justify a growing disengagement from research by the pharmaceutical industry [160]. If not counterbalanced by broad and dedicated investment in SMEs and universities, this process might be highly detrimental to the already damaged social contract between this industry and society in the short and medium term [161].

We believe that homology modeling, docking and virtual screening can be successfully combined to accelerate drug discovery. Just as it was the case for drugs discovered at the beginning of the 20^th^ century, an important part of the reality will always remain hidden, but the synergy of these techniques is part of the ‘New Deal’ we advocate. With evidence-based SBDD scientists will be put back in control, and they will engage their minds again, taking pride in their “craft”.

## Figures and Tables

**Table:** 

ADME/tox	absorption, digestion, metabolism, excretion, and toxicity
ATP	adenosine triphosphate
β2AR	beta-2 adrenergic receptor
Cα	alpha carbon
CoMFA	comparative molecular field analysis
CoMSIA	comparative molecular similarity index analysis
CXCR4	C-X-C chemokine receptor type 4
ECL2	extracellular loop-2
Erk2	Extracellular signal-regulated kinases 2
FBDD	fragment-based drug design
GPCR	G-protein coupled receptor
HIV	human immunodeficiency virus
HTS	high throughput screening
IC50	half maximal inhibitory concentration
ITC	isothermal titration calorimetry
JCAMD	Journal of Computer-Aided Molecular Design
Kd	dissociation constant
MDS	molecular dynamics simulation
MlogP	Moriguchi octanol-water partition coefficient
NR	nuclear receptor
PK	protein kinase
PSA	polar surface area
QSAR	quantitative structure-activity relationship
SBDD	structure-based drug design
RNA	ribonucleic acid
SME	small to medium enterprise
tRNA	transfer RNA
TGT	tRNA-guanine transglycosylate
VLS	virtual ligand screening
